# Development of an Efficient Targeted Cell-SELEX Procedure for DNA Aptamer Reagents

**DOI:** 10.1371/journal.pone.0071798

**Published:** 2013-08-13

**Authors:** Susanne Meyer, John P. Maufort, Jeff Nie, Ron Stewart, Brian E. McIntosh, Lisa R. Conti, Kareem M. Ahmad, H. Tom Soh, James A. Thomson

**Affiliations:** 1 Department of Molecular, Cellular and Developmental Biology, University of California Santa Barbara, Santa Barbara, California, United States of America; 2 Morgridge Institute for Research, Madison, Wisconsin, United States of America; 3 Department of Cell and Regenerative Biology, University of Wisconsin School of Medicine and Public Health, Madison, Wisconsin, United States of America; 4 Department of Materials, University of California Santa Barbara, Santa Barbara, California, United States of America; 5 Department of Mechanical Engineering, University of California Santa Barbara, Santa Barbara, California, United States of America; 6 Interdepartmental Program in Biomolecular Science and Engineering, University of California Santa Barbara, Santa Barbara, California, United States of America; Northwestern University, United States of America

## Abstract

**Background:**

DNA aptamers generated by cell-SELEX offer an attractive alternative to antibodies, but generating aptamers to specific, known membrane protein targets has proven challenging, and has severely limited the use of aptamers as affinity reagents for cell identification and purification.

**Methodology:**

We modified the BJAB lymphoblastoma cell line to over-express the murine c-kit cell surface receptor. After six rounds of cell-SELEX, high-throughput sequencing and bioinformatics analysis, we identified aptamers that bound BJAB cells expressing c-kit but not wild-type BJAB controls. One of these aptamers also recognizes c-kit endogenously expressed by a mast cell line or hematopoietic progenitor cells, and specifically blocks binding of the c-kit ligand stem cell factor (SCF). This aptamer enables better separation by fluorescence-activated cell sorting (FACS) of c-kit^+^ hematopoietic progenitor cells from mixed bone marrow populations than a commercially available antibody, suggesting that this approach may be broadly useful for rapid isolation of affinity reagents suitable for purification of other specific cell types.

**Conclusions/Significance:**

Here we describe a novel procedure for the efficient generation of DNA aptamers that bind to specific cell membrane proteins and can be used as high affinity reagents. We have named the procedure STACS (Specific TArget Cell-SELEX).

## Introduction

There is an ongoing need in basic biological research, clinical diagnostics and therapeutics for affinity reagents that can target proteins on the surface of mammalian cells with high specificity. Monoclonal antibodies continue to be predominantly used for these purposes. However, production of monoclonal antibodies in large quantities is time-consuming and expensive, and there is demand for a high-throughput and low-cost method for generating affinity reagents. This is particularly true for the emerging fields of proteomics and biomarker discovery, which are heavily dependent on the large-scale generation of high-quality affinity reagents [1].

The past 20 years have witnessed growing interest in aptamers as alternative affinity reagents. Aptamers are short DNA or RNA oligonucleotides that have many intrinsic advantages over antibodies. They are chemically synthesized, easily modified and thermostable. Aptamers can also achieve very high target affinity–in the pico-molar range, comparable to those attainable with antibodies [2]. Aptamers are derived from random oligonucleotide pools through a process known as SELEX (Systematic Evolution of Ligands by EXponential enrichment), which involves repetitive rounds of partitioning and enrichment and is most commonly performed with purified target proteins immobilized on beads[3]–[5]. This approach suffers from a significant drawback in that many important protein targets such as cell surface receptors are extremely difficult to purify. Even those that can be successfully purified may not retain their native conformation when immobilized, such that selected aptamers may not recognize the natural structure of proteins as expressed on living cells [6], [7].

As an alternative to selecting against purified proteins on beads, one may select for proteins expressed on the surface of whole cells in a process called cell-SELEX [8], [9]. Cell-SELEX is commonly used to identify cancer cell-specific affinity reagents and biomarkers, but the specific targets usually remain undefined[2], [9]–[14]. Cerchia et al. reported a differential cell-SELEX procedure yielding aptamers that preferentially bind to tumorigenic cancer cell lines [15]. This group also first described cell-SELEX using engineered cell lines expressing mutant receptors [16]. After fifteen rounds of selection, Cerchia et al. analyzed the binding activity of their aptamer pools and identified specific binding sequences by traditional cloning technique. The Giangrande group further optimized cell-based selections and combined RNA aptamer cell-SELEX with high throughput sequencing to discover internalizing RNA aptamers to vascular smooth muscle cells [17]. The same group recently published the identification of internalizing RNA aptamers using a rat Her2 transgenic mouse mammary carcinoma model [18].

However, to date targeted cell-SELEX procedures based on the general use of engineered cell lines over-expressing specific protein targets have been challenging. To address this issue, we have developed a method called STACS (Specific TArget Cell Selex) that incorporates specific cell surface protein expression in a lymphoblastoma cell line, cell-SELEX, high throughput sequencing and bioinformatic analysis. By combining these individual processes, we can generate aptamers against cell-surface proteins rapidly and efficiently. Because we are primarily interested in generating aptamer reagents for isolating specific stem and precursor cell populations, we have applied STACS to identify a DNA aptamer that binds to the murine c-kit receptor, one of the key markers used in the isolation of hematopoietic stem cells [19], [20]. By stably over-expressing c-kit on a lymphoblastoma cell line (BJAB) that grows in suspension culture, we essentially make living cells the “bead,” avoiding time-consuming protein purification while also presenting the target receptor in a more natural form. After only three weeks and six rounds of STACS, we identified aptamer pools that contained c-kit-specific binders as confirmed by flow cytometry. By applying high-throughput sequencing and custom bioinformatics analysis, we isolated a high-affinity c-kit aptamer that specifically identifies c-kit positive cells in a heterogeneous mixture of murine bone marrow cells, which we demonstrate can be used as a cell sorting reagent.

## Results

### STACS Method Overview

**Figure 1:**
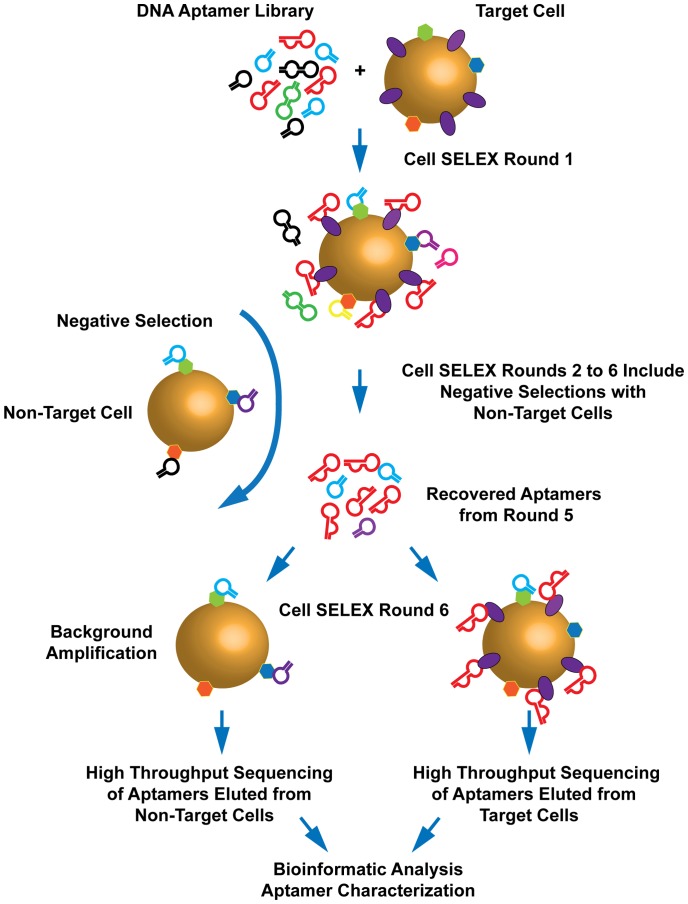
The STACS procedure. Our method has five essential steps: generation of a target-expressing cell line, five or six rounds of cell-SELEX, characterization of fluorescence-labeled aptamer pools by FACS, high-throughput sequencing, and bioinformatics and characterization of individual aptamer sequences by flow cytometry.

The individual steps of STACS are outlined in Figure 1. We used c-kit-expressing BJAB target cells, and performed six rounds of cell-SELEX with a DNA aptamer library. After the first round, we included a negative selection step with parental BJAB non-target cells to reduce background binders. In round 6, the selected aptamer pool was incubated in parallel on non-target and target cells. We eluted the aptamer pools from both cell lines and subjected them to high-throughput sequencing and informatic analysis, identifying individual candidate aptamer sequences based on their enrichment ratios in target versus non-target cell pools. These candidates were then further characterized by flow-cytometry.

### Generation of a c-kit-Expressing Lymphoblastoma Cell Line

**Figure 2:**
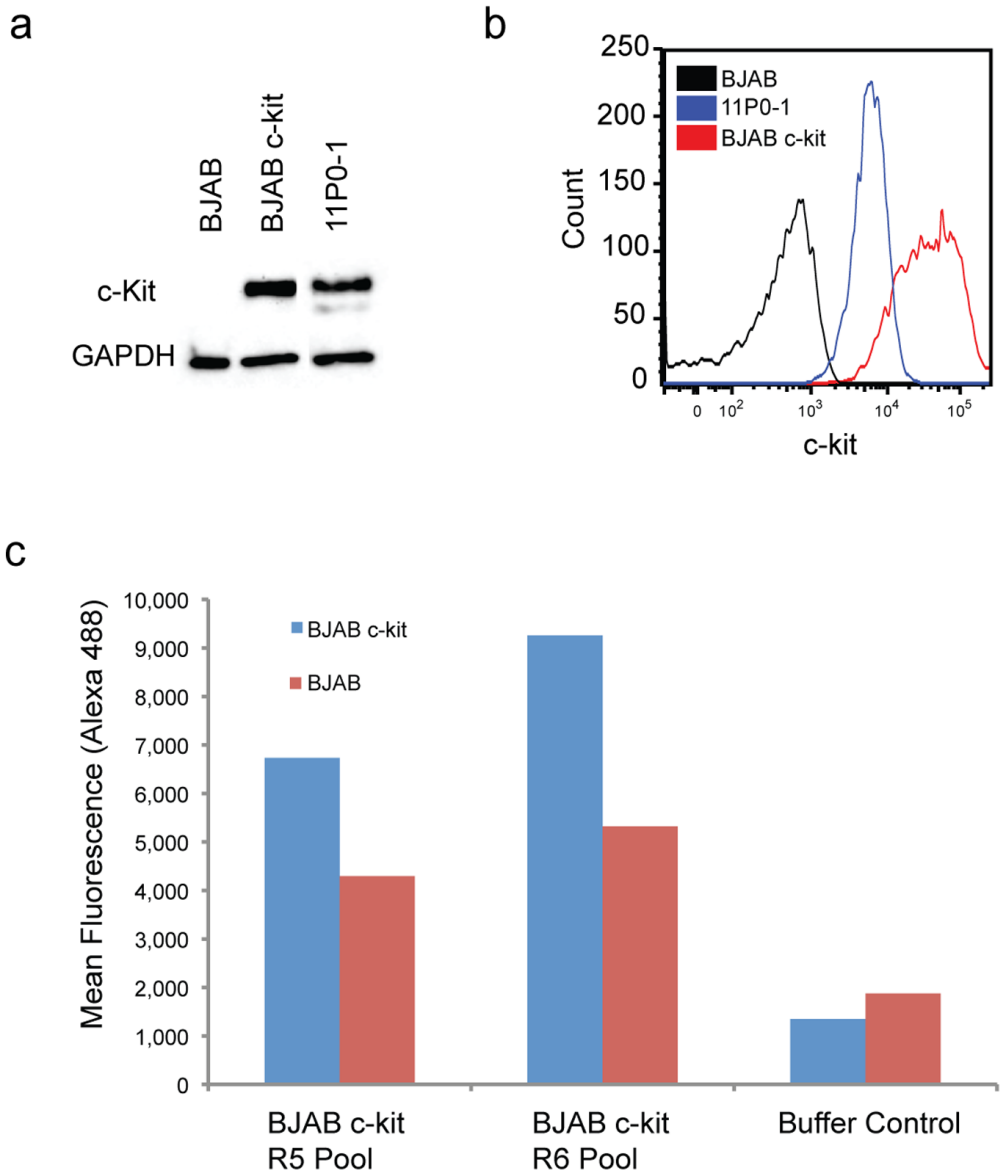
C-kit expression on cell lines and testing of R5 and R6 aptamer pools. The BJAB c-kit cell line stably expresses the murine c-kit cDNA. Shown here are (a) Western blot and (b) FACS analysis of c-kit expression for parental BJAB cells, 11P0-1 mouse mast cells and BJAB c-kit cells. **(c)** We tested binding with 200 nM samples of single-stranded R5 and R6 aptamer pools from BJAB-c-kit cell-SELEX via flow cytometry. Blue bars represent mean fluorescence values of aptamer pools binding to BJAB c-kit cells. Red bars indicate binding to BJAB parental cells.

BJAB is a lymphoblastoma cell line that is grown in suspension, eliminating the need for dissociation reagents that can cleave extracellular target proteins. Suspension cells also enable high-stringency washes, which are important for reducing background binders. We generated BJAB c-kit target cells by stably expressing the murine c-kit cDNA through piggyBAC™ insertion [21]. This rapid procedure typically requires less than two weeks. To isolate the highest expressers, we sorted single cells by FACS based on c-kit expression. We expanded twelve clones and chose one high c-kit expressing clone for further analysis. Western blot and FACS data (Fig. 2a, b) confirmed high c-kit expression in this clone. Compared to the parental BJAB cell line, which has no c-kit expression, BJAB c-kit cells exhibited high levels of c-kit expression, with an over 100-fold higher mean fluorescence signal in the FACS results (Fig. 2b). As a positive control for normal c-kit expression, we selected the 11P0-1 mouse mast cell line, which showed lower c-kit expression compared to the BJAB c-kit cell line (Fig. 2a, b).

### Cell-SELEX

We performed six rounds (R1–6) of cell-SELEX with BJAB c-kit cells using a DNA library where each member consists of a 29-nucleotide (nt) variable region flanked by 20-nt PCR primer-binding regions. Our selection strategy involved increasing the selection pressure from R2 onward by reducing the cell number and increasing the stringency of the washes (see online methods for details). To remove nonspecific binders from the pools, we performed negative selection with parental BJAB cells from R2–6. It is important to note that the BJAB cell line used for negative selection has no endogenous c-kit expression. We demonstrated enrichment of c-kit-specific binders in R5 and R6 via flow cytometry (Fig. 2c). For R6, the aptamer pool was split. Half was selected against BJAB c-kit, the other half was selected against wild type BJAB, and both pools were separately sequenced. Aptamers for subsequent binding analysis were selected based on their increased representation in the R6 BJAB c-kit pool relative to the wild type pool.

### High-Throughput Sequencing and Bioinformatics

High-throughput sequencing is a critical component of our cell-SELEX method because it enables us to identify protein target-specific aptamers after only six rounds of selection. We performed high-throughput sequencing of the R6 aptamer pools, which were eluted from either c-kit-expressing or parental BJAB cells. We generated an average of 37 million raw sequence tags per pool, including total numbers of both forward and reverse sequence reads in each pool. We matched sequenced forward tags to their reverse complement tags, resulting in an average of 18.5 million aptamer sequence tags per pool (see Table 1).

**Table 1:** Analysis of sequences in R6 aptamer pools.

Aptamer pool	Number of RawSequence tags	Number of tagspassing forwardprimer filtering	Number of tagspassing reverseprimer filtering	Passingrate	Number ofcorrectsequence tags	Number ofunique sequencetags	Percent duplicatesequences
**BJAB c-kit Round 6**	36,882,646	17,137,967	17,462,991	94%	34,600,958	22,809,211	34.07
**BJAB Round 6**	38,535,156	18,049,867	18,064,964	94%	36,114,831	31,167,387	13.69

Numbers of raw, corrected and unique sequence tags are listed as well as the percentage that passed filtering and percent duplicate sequences.

Informatic analysis showed that the number of unique sequence tags was lowest and the percentage of duplicates was highest in the BJAB c-kit R6 pool, indicating enrichment of target-specific sequences in this round (see Table 1). To identify c-kit-specific binders, we calculated the enrichment ratios of aptamer tag numbers in the BJAB c-kit R6 pool over the parental BJAB R6 pool. We sorted all sequence tags by these ratios, and the 20 aptamers with the highest ratios are listed in Table 2. We chose the top seven aptamers from these 20 based on their high enrichment ratios but also included three additional aptamers that had high tag counts in the BJAB c-kit R6 pool. By applying custom bioinformatics quality control, we ensured that only aptamers of the correct length and containing both flanking constant regions were considered for further characterization (see Materials and Methods) Clustal Omega was used to cluster the top 20 aptamer sequences (as chosen by enrichment ratio) (See Fig. S1).

**Table 2:** Top 20 c-kit aptamer sequences isolated via STACS (boldface indicates aptamers chosen for further analysis).

Top 20	Sequence	BJAB R5	BJABc-kit R5	BJAB R6	BJABc-kit R6	BJAB c-kitR6/BJAB R6	AptamerName
**1**	**GTGTGTACATTCTCTTCGTTTGCCTTGAC**	**4**	**5**	**11**	**2662**	**242**	**Kit-242**
**2**	**AATTCAGTCAGGTAGGGTAGGATATGTGG**	**NA**	**3**	**1**	**156**	**156**	**Kit-156**
**3**	**TGTTTACATTCTCTTCGTTTGCATGTGCG**	**3**	**7**	**3**	**464**	**155**	**Kit-155**
**4**	**GGTGTTTACATTCTCTTCGTTTGCGTTGA**	**18**	**29**	**20**	**3068**	**153**	**Kit-153**
**5**	**GGTGTTTACATTCTCTTCGTTTGCATTGA**	**12**	**9**	**18**	**2711**	**151**	**Kit-151**
**6**	**GCTCAACGCGGGACGGCTCTCCCATTGAC**	**6**	**16**	**16**	**2069**	**129**	**Kit-129**
**7**	**TGTTGACATTCTCTTCGTTTGCATCTGCG**	**2**	**8**	**4**	**436**	**109**	**Kit-109**
8	GGTGTTTCCTTTCCCTTCGTTTGCCTTGG	1	1	1	107	107	Kit-107
9	AATTTAGTCAGGTAGGGTAGGATATGTGG	4	2	2	204	102	Kit-102
**10**	**GTGTATACATTCTCTTCGTTTGCCTTGAC**	**21**	**16**	**37**	**3448**	**93**	**Kit-93**
**11**	**TGTTAACATTCTCTTCGTTTGCATCTGCG**	**5**	**2**	**6**	**536**	**89**	**Kit-89**
12	TGTTAACATTCTCTTCGTTTGCATCACTA	NA	NA	NA	84	85	Kit-85
13	GTGTTTACATTCTCTTCGTTTGCCGCTGG	2	1	1	84	84	Kit-84
14	GTGTATACATTCTCTTCGTATGCCGCCTT	NA	1	NA	77	78	Kit-78
15	GTGTATACATTCTCTTCGTTTGCCTCTGG	NA	1	1	77	77	Kit-77.1
16	AATTGAGTCAGGTAGGGTAGGATAAGTGG	NA	4	3	230	77	Kit-77.2
**17**	**GCTCTACGCGGGACGGCTCTCCCAGTGAC**	**28**	**14**	**25**	**1902**	**76**	**Kit-76**
18	GTGTATACCTTCTCTTCGTTTGCCTCCGG	NA	NA	1	76	76	Kit-76.2
19	GTTGTAATAGGTTGGGTGGGTGCAACCG	NA	1	1	76	76	Kit-76.3
20	GTTTTTACATGCTCGTCGTTTGCCTCCGG	3	NA	1	73	73	Kit-73

### Aptamer Characterization by Flow Cytometry

**Figure 3:**
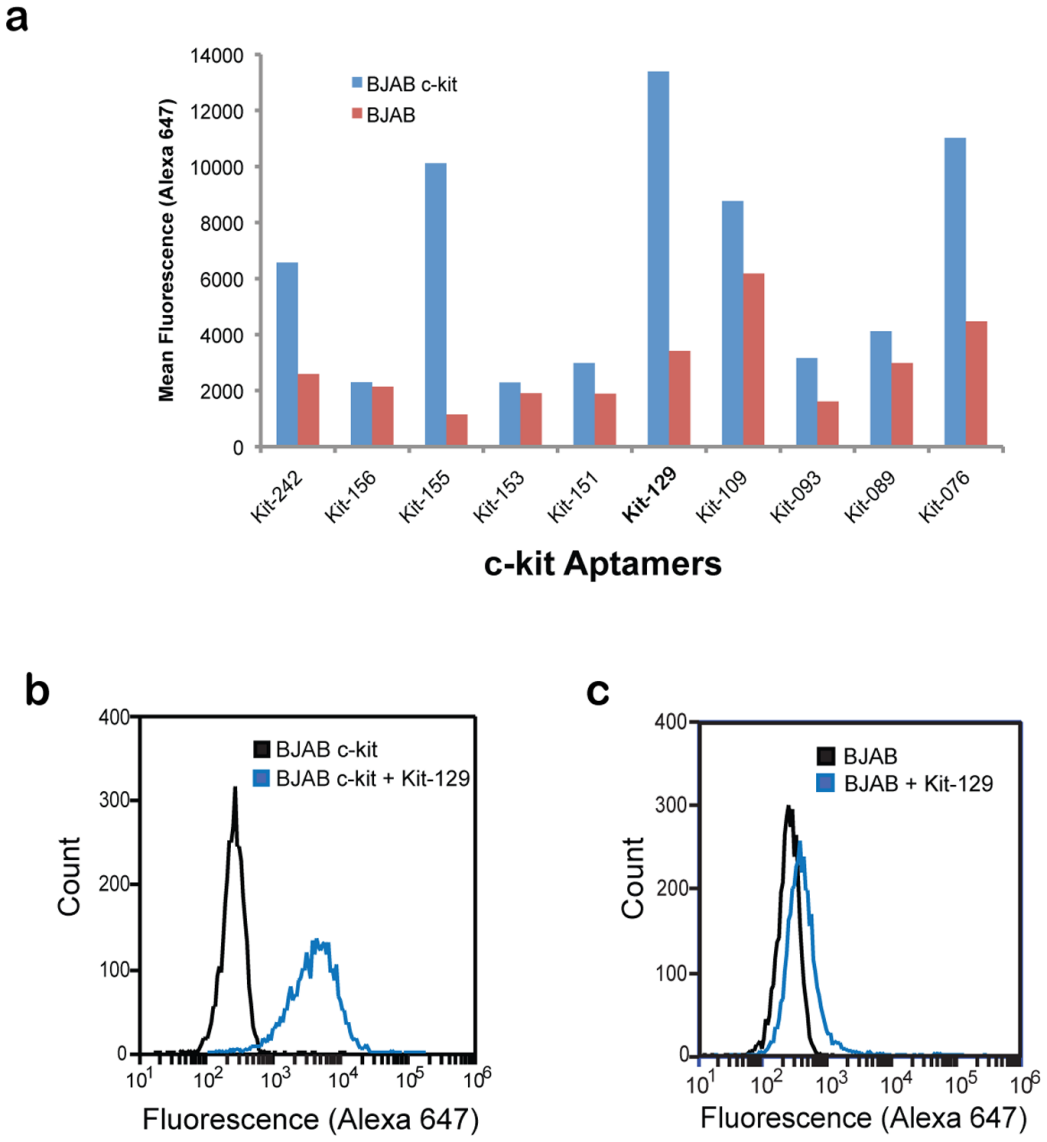
Characterization of c-kit aptamer binding by flow cytometry. (a) Mean fluorescence values from duplicate binding experiments with 10 c-kit aptamers tested at 100 nM with BJAB c-kit (blue) or BJAB parental (red) cells. FACS analysis with 50 nM Kit-129 tested on (b) BJAB c-kit cells and **(c)** BJAB parental cells.

**Figure 4:**
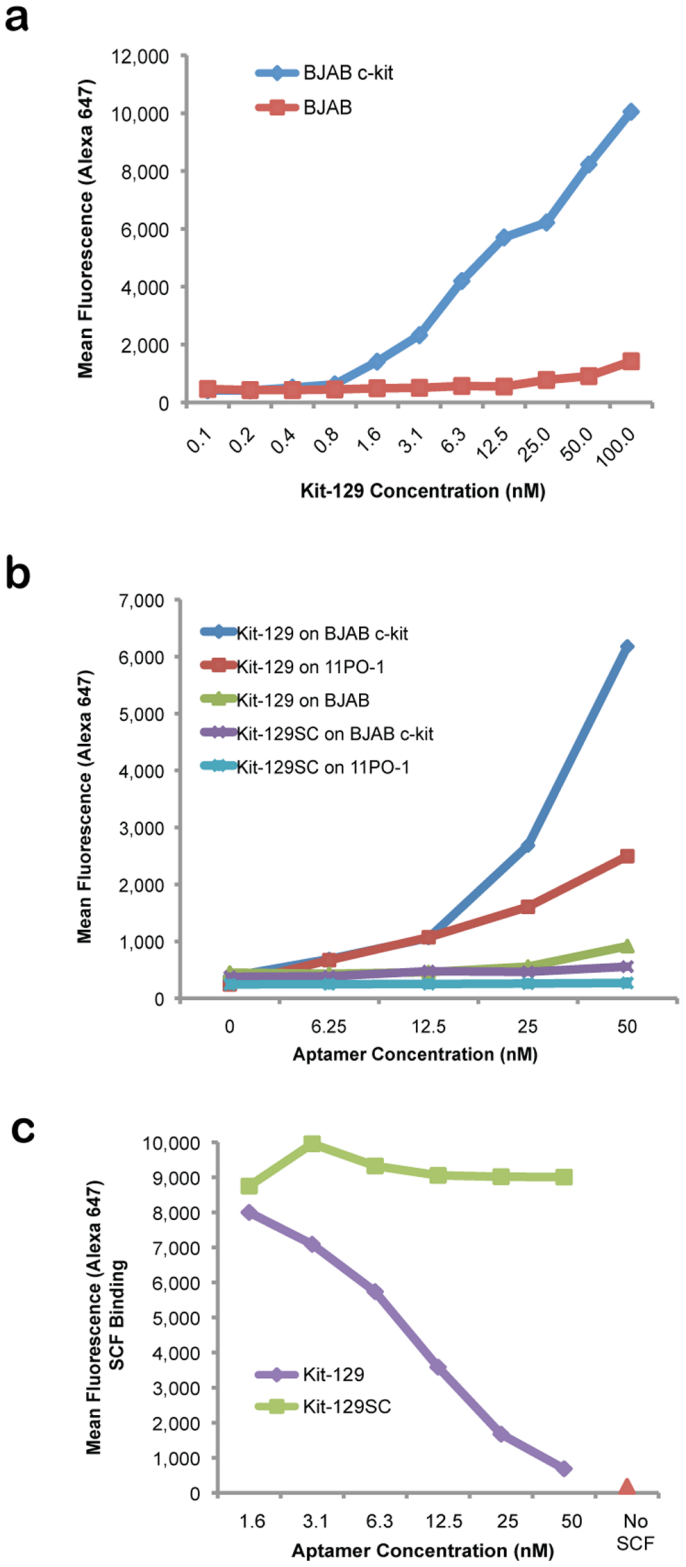
Characterization of Kit-129 binding and ligand-blocking activity. (a) Kit-129 has high specificity for BJAB c-kit cells. Shown are mean fluorescence values of Alexa-647-labeled Kit-129 aptamer tested in serial dilutions from 0 to 100 nM by flow cytometry. (b) Alexa-647-labeled Kit-129 binds to mouse mast cell line 11P0-1 (which naturally expresses c-kit) as well as BJAB c-kit cells, while a scrambled version of this aptamer (Kit-129SC) does not. (c) Kit-129 blocks SCF binding to c-kit expressed by BJAB cells. Serial dilutions of Kit-129 or Kit-129SC were added to either cell line followed by addition of biotin-labeled SCF and streptavidin-Alexa-647 conjugate. SCF binding was measured by flow cytometry.

Our goal was to identify a sorting reagent for c-kit positive cells. We tested the ten selected c-kit aptamers for specific binding to BJAB c-kit cells via flow cytometry (Fig. 3a). The Kit-129 aptamer showed highest specificity for BJAB c-kit cells (Fig. 3b) compared to parental BJAB cells (Fig. 3c, p = 0.000061). We tested Kit-129 in serial dilutions from 0 to 100 nM, and found that this aptamer displayed high affinity for target cells with low background binding to parental BJAB cells (Fig. 4a). The K_d_ of Kit-129 is 12.21 nM (Fig. S2).

**Figure 5:**
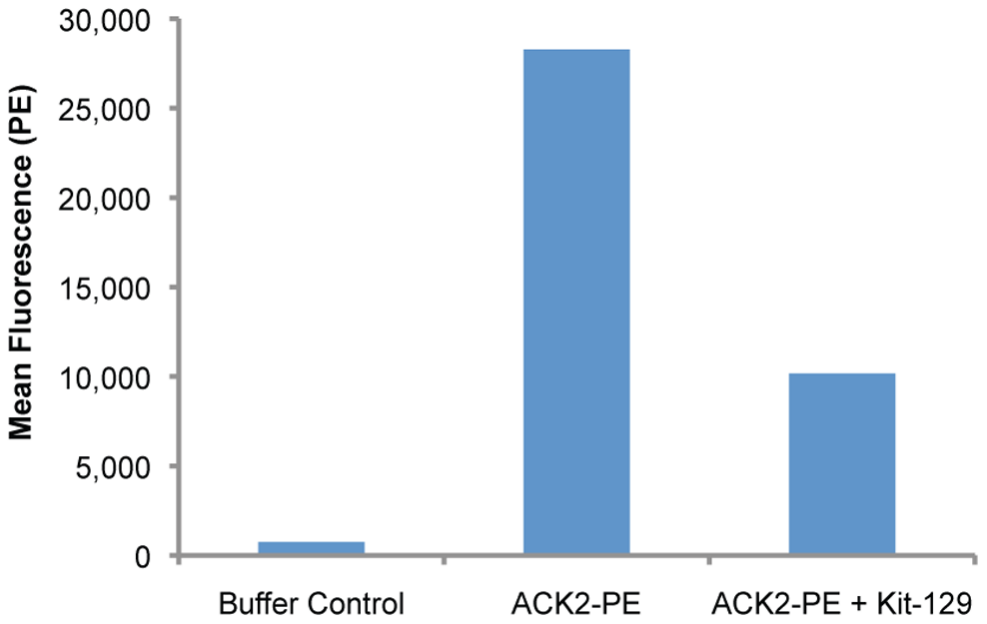
Kit-129 competes with ACK2 in binding to BJAB c-kit cells. Buffer or Kit-129 aptamer and phycoerythrin (PE)-labeled anti-c-kit antibody ACK2 were added to BJAB-c-kit cells and incubated for 1 hour on ice. ACK2-PE binding was measured by flow cytometry.

Kit-129 also bound to endogenously expressed c-kit on 11P0-1 cells. Consistent with target protein levels, the mean fluorescence signal for binding to 11P0-1 was lower compared to BJAB c-kit cells (Fig. 4b). We ruled out non-specific binding using a scrambled aptamer of the same base composition (Kit-129SC), which did not bind to 11P0-1 or BJAB c-kit. We further demonstrated aptamer specificity as well as functionality in a competition assay with stem cell factor (SCF), which is the ligand for c-kit. Kit-129 but not Kit-129SC blocked SCF binding to BJAB c-kit cells in a dose-dependent fashion (Fig. 4c). A competition assay with the c-kit binding antibody ACK2 further confirmed specificity for c-kit protein (Figure 5).

**Figure 6:**
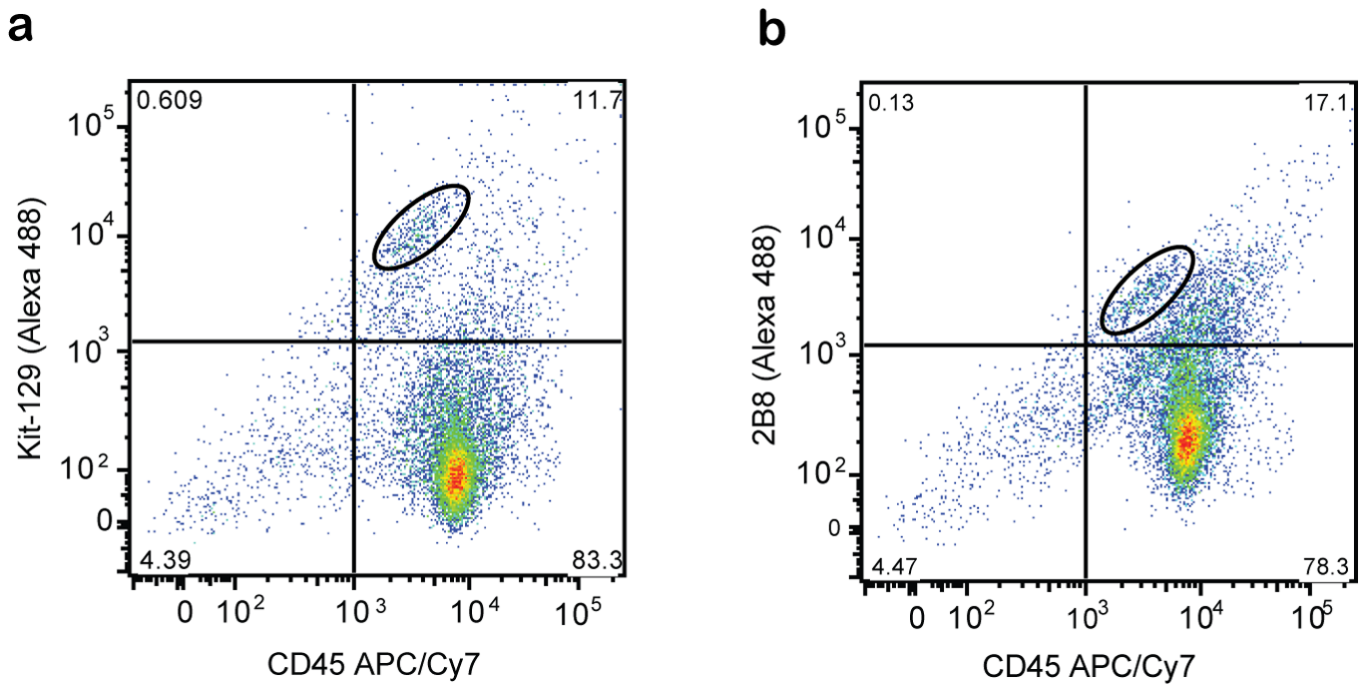
Identification of c-kit expressing bone marrow cells. Double labeling of mouse bone marrow cells with anti-CD45 antibody and (a) Kit-129 aptamer or (b) rabbit anti-c-kit antibody 2B8.

**Figure 7:**
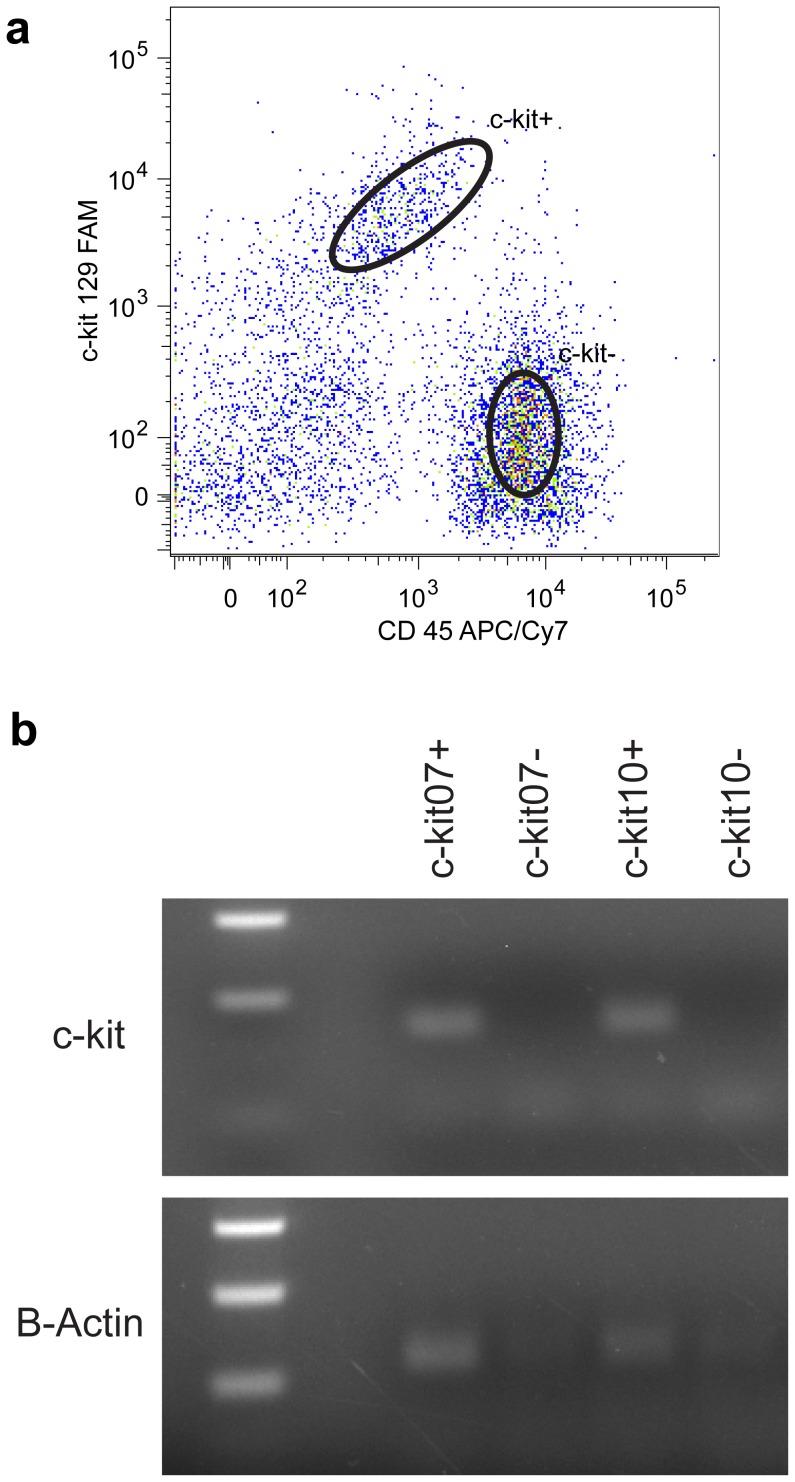
Verification of c-kit expression in aptamer sorted bone marrow cells. Mouse bone marrow cells were double labeled with anti-CD45 and Kit-129 aptamer. Kit-129 positive and negative populations were sorted (A) and qPCR performed to analyze c-kit expression within each population. The CT value for the c-kit positive population was 27.3 while c-kit mRNA was undetectable even after 40 cycles for the c-kit negative population. ActinB was used as an internal control and c-kit expression data was normalized to ActinB. (B) Products from qPCR analysis run on a 2% agarose gel.

To determine if DNA aptamers specific to c-kit would be suitable for cell purification, we labeled mouse bone marrow with the Kit-129 aptamer. We collected whole bone marrow and co-labeled the cells with an antibody against CD45, a marker widely present on hematopoietic cells, and either Kit-129 or an antibody for mouse c-kit (2B8) (Fig. 6a,b). Both Kit-129 and 2B8 labeled 10 to 20% of the white blood cell population, demonstrating the usefulness of the aptamer as a sorting reagent (Fig. 6a,b). Notably, there was approximately 10-fold better separation between the double-positive populations in the aptamer- versus antibody-labeled bone marrow cells, indicating that the aptamer represents a superior sorting reagent (Fig. 6a,b). Additionally, c-kit positive bone marrow cells sorted with c-kit aptamers expressed c-kit mRNA (qPCR CT = 27.3), while negative populations had no detectable mRNA c-kit expression by 40 qPCR cycles, further demonstrating the specificity of the reagent in a complex cellular context (Fig. 7).

## Discussion

We present STACS, a new cell-SELEX method that allows efficient generation of specific DNA aptamer reagents against full-length, cell surface-expressed protein targets. In this report we used the murine c-kit receptor as a target. After six rounds of selection, we identified a highly specific DNA aptamer reagent with superior qualities for cell sorting compared to a commonly used anti-c-kit antibody. This aptamer allows for excellent separation of a c-kit-positive subpopulation in a complex mixture of bone marrow cells, and thus functions as a high quality FACS reagent. This c-kit aptamer is an example of a rapid, cost effective strategy for producing clinically relevant reagents.

The STACS method has many important advantages. To achieve stable target protein expression in a suspension cell line, we used the PiggyBac™ cDNA delivery system. PiggyBac™ is a non-viral, transposon-based system that can carry cargo of up to 14.3 kb and facilitates expression of full-length receptors and other large trans-membrane proteins in mammalian cells [22]. Suspension cell lines such as BJAB are ideal for cell-SELEX, since they allow high stringency washes and eliminate the need for potentially damaging enzymatic dissociation treatment. An important detail of STACS is that the last round aptamer pools are selected in parallel on both parental and target protein-expressing cells. Negative cell-based selection is an important aspect of successful cell-SELEX as demonstrated in RNA-aptamer cell-SELEX [17]. To retain any rare specific binders we PCR amplified the entire eluted round 1 pool and performed negative selections starting in round 2, as previously described [12]. Once the cell-SELEX rounds are completed, selected aptamers are characterized via high-throughput sequencing and custom bioinformatic analysis, which includes calculating the enrichment ratios of aptamer tag numbers in target expressing BJAB pool over parental BJAB pool. Thiel et al. [17] also utilized a selective enrichment strategy to predict binders, tracking an increase in duplicates and a decrease in library complexity over the rounds. In Thiel et al. [17], however, library complexity was measured via a DNA melting assay and sequencing, which differs from our approach of much deeper sequencing. DNA melting assay was not able to detect a decrease in complexity in late rounds while relatively shallow sequencing did detect this decrease, suggesting that deep sequencing may be a more accurate method for assessing library complexity. In addition, Thiel et al. used measures of aptamer presence across rounds, cluster size, edit distance, and predicted structure to assist in predicting binders and determining aptamer families [17]. In our demonstration of STACS, we sequenced complete aptamer pools and conducted bioinformatic analysis on a total of 34 million sequences (see Table 1). These techniques enabled us to select relatively rare aptamers with desired properties while also identifying and excluding less desirable dominant sequences.

STACS improves upon current cell-SELEX strategies with regard to time requirements and target specificity and in cost of materials and labor. It allows identification of target-specific aptamers in six selection rounds, which is superior to a recently described cell-SELEX method by FACS sorting requiring ten rounds [23]. Most traditional cell-SELEX procedures take two to three months to complete and involve 12–20 rounds of selection [24]. It has been reported that performing more than 12 selection rounds reduces the probability of identifying high affinity aptamers, mostly because PCR artifacts can dominate the population of aptamers in the enriched pool [23], [25]. Furthermore, true high-affinity binders are often missed because of the limitations of traditional aptamer cloning techniques, wherein only a limited number of clones (∼100) can be isolated and sequenced.

Although our STACS method is already highly optimized, we see several possibilities for improvement. With the goal of further reducing the time and labor required, we plan to perform HTS and more detailed informatic analysis of aptamer pools at earlier selection rounds. We have recently shown that high-throughput sequencing and bioinformatics make it possible to discover specific, high-affinity aptamers for purified, bead-immobilized proteins after only three rounds of selection [12], [26], and others have described identification of high-affinity aptamers against purified proteins by HTS after five rounds of selection [25]. Therefore, we will evaluate the effectiveness of performing HTS and informatic analysis at R3–5. Furthermore, cluster analysis will enable us to screen for specific aptamers with unique sequences even more systematically. HTS-guided aptamer truncations might further improve aptamer characteristics and enable even more cost-effective synthesis. Finally, multivalent aptamers could be synthesized and tested for functional properties [25].

In conclusion, even without any further modifications, our new method is a powerful and novel procedure that addresses the need for high-quality reagents that recognize challenging cell-surface targets with high affinity in both basic research and therapeutics. In the field of developmental biology for example, many cell differentiation studies have restricted their focus to the characterization of RNA expression levels. However, since cDNAs are now commercially available for most genes, the generation of aptamer affinity reagents for developmentally-relevant proteins via STACS will open vast new research avenues.

## Materials and Methods

### Cell Maintenance

We cultured BJAB cells, a human Burkitt’s lymphoma cell line (gift from Dr. William Sugden, UW Madison) in RPMI 1640 medium (Invitrogen) supplemented with 10% fetal bovine serum (FBS, American Tissue Culture Collection; ATCC) [27]. BJAB cells expressing murine c-kit were maintained in BJAB culture medium with 1 µg/ml puromycin (Gibco). Mouse mast cell line 11P0-1 (ATCC) was cultured in RPMI 1640 medium supplemented with 0.05 mM 2-mercaptoethanol and 10% fetal bovine serum.

### Generation of c-Kit-Expressing BJAB Cell Line

BJAB cells expressing c-kit were generated by piggyBac™ (Systems Biosciences) insertion of the murine c-kit cDNA [21]. The expression cassette was created by cloning the murine c-kit cDNA behind the EF1α promoter followed by an internal ribosome entry site (IRES) and the puromycin-resistance gene for selection purpose. The cassette was inserted between flanking sequences of the 3′ and 5′ piggyBac™ terminal repeats. The resulting expression plasmid was co-transfected with piggyBac™ transposase RNA into BJAB cells using the Amaxa electroporation system (Lonza). Following selection with 1 µg/ml puromycin, cells were analyzed by flow cytometry and the highest expressing cells were sorted as single cells into a 96-well plate. Twelve clones were picked for expansion and characterized for c-kit expression by Western blot and flow cytometry. One BJAB clone expressing high levels of c-kit was picked for further experiments.

### Flow Cytometric Analysis of c-kit Expressing BJAB Cells

BJAB clones were spun down, washed, and re-suspended in Hanks Buffered Saline Solution (HBSS; Life Technologies) with 2% FBS. The cells were then blocked in HBSS/2%FBS/2% rat serum (Gibco) for 10 min and washed. Clones were labeled for 30 min with either clone ACK2 or 2B8 anti-c-kit antibody (Novus Biologicals) diluted 1∶100 in HBSS with 2% FBS. Cells were then analyzed for c-kit expression on the FACSCanto II cytometer (BD Biosciences).

### Western Blot Analysis of c-kit Expressing BJAB Cells

Total protein was collected by cell lysis in RIPA buffer composed of 50 mM Tris pH7.9 (Fisher), 150 mM NaCl (Fisher), 1 mM EDTA (Fisher), 1% Triton X-100 (Sigma), 0.5% sodium deoxycholate (Sigma), 0.5% SDS (Sigma), 25 units/ml benzonase) (Novagen) and protease and phosphatase inhibitors (Roche), followed by sonication in ice water for 150 seconds in 15-second intervals. Proteins concentrations were determined by measuring absorption at 230 nm. Equal protein amounts were run on a 4–15% polyacrylamide gel, verified by loading control. The proteins were transferred to nitrocellulose membranes, which were then blocked for 1 hour in TBS-T buffer containing 137 mM sodium chloride, 20 mM Tris, 0.1% Tween-20 pH 7.6 and 5% milk. Blots were incubated with anti-c-kit (D13A2) XP antibody (Cell Signaling) diluted 1∶1000 overnight at 4°C in TBS-T/5% BSA, washed with TBS-T, and then incubated with anti-rabbit-horseradish peroxidase antibody conjugate (Cell Signaling) diluted 1∶1000 in TBS-T/5% milk for 1 hour. Blots were developed and visualized with the Fujifilm LAS 4000 imager.

### Cell SELEX with DNA Aptamer Library

A recently published protocol for cell-SELEX was used as a starting point for developing our cell-SELEX procedure [12]. We used a random DNA aptamer library of molecules comprising a 29-nt variable region flanked by two fixed 20-nt primer-binding sites (5- AGC AGC ACA GAG GTC AGA TG - 29N - CCT ATG CGT GCT ACC GTG AA -3), which was synthesized by Integrated DNA Technologies (IDT). For the first cell-SELEX round, we diluted 4 nmol of DNA aptamer library into SELEX buffer composed of Dulbecco’s phosphate-buffered saline with calcium and magnesium (DPBS; Invitrogen) plus 5 mM MgCl_2_, 0.45% glucose and 100 mg/ml tRNA. We heated the library for 10 min at 95°C, followed by snap cooling on ice for 10 min, and then mixed the library with 5.0×10^6^ BJAB c-kit cells in SELEX buffer containing 0.1% BSA (Sigma) in a 1.5 ml tube. We incubated library and cells for 1 h rotating at 4°C, and then removed unbound DNA by centrifugation and two washes in SELEX buffer with 0.1% BSA in 1.5 ml tubes. We eluted the bound DNA by heating the cells in distilled water for 10 min at 95°C then collecting the supernatant after a 5 min spin at 13,100×*g*.

In R1 of cell-SELEX, we pre-amplified the eluted DNA via seven PCR cycles using GoTaq PCR mix (Promega), forward primer (5- AGC AGC ACA GAG GTC AGA TG -3) and reverse primer (5- ACG GTA GCA CGC ATA GG -3). The collected PCR product was then subjected to pilot PCR to determine the optimal PCR cycle number. Alexa-488-labeled forward primers and phosphate-labeled reverse primers (IDT) were used in subsequent PCR amplifications to generate fluorescently-labeled single-stranded DNA pools for later testing. For this first round of cell-SELEX, PCR products were amplified with another six rounds of PCR. Double-stranded DNA was purified using the Qiagen PCR purification kit. Single-stranded DNA was generated by lambda exonuclease (New England Biolabs) digestion, followed by phenol extraction and ethanol precipitation [28]. The resulting purified aptamers were run on a 4.5% agarose gel (Sigma Aldrich) to confirm correct DNA size.

In R2–6 of cell-SELEX, we included negative selection with wild-type BJAB cells to eliminate non-specific background binders. In brief, we first performed positive selection with BJAB c-kit cells, then eluted the bound DNA by heating for 10 min at 95°C with SELEX buffer. We collected the eluted DNA, snap-cooled it on ice and incubated it for 1 h rotating at 4°C with 8.0×10^6^ wild-type BJAB cells in SELEX buffer with 0.1% BSA. After centrifugation, we discarded the cell pellet and collected the negatively selected DNA in the supernatant for processing by pilot PCR, full PCR, and double-stranded and single-stranded DNA purification as described above. For R2–6, we increased the selection pressure by lowering BJAB c-kit cell numbers from 5.0×10^6^ cells to 4.0×10^5^ cells and at the same time decreasing the incubation time from 1 hour to 15 min, while increasing the number of washes from two to three. For R6 we performed cell-SELEX in parallel on BJAB c-kit cells and wild-type BJAB cells, and used the resulting isolates for subtractive DNA sequence analysis.

### High Throughput DNA Sequencing and Analysis of R5 and R6 Pools

We adapted previously-described methods for high-throughput sequencing of DNA aptamer pools to suit this project [26]. Briefly, we used the TruSeq DNA sample preparation kit v.2 (Illumina) to prepare the double-stranded aptamers for sequencing on the HiSeq 2000 (Illumina). We initially prepared each sample from 136 ng of DNA, which were subjected to end repair, 3′ addition of adenosine, adapter ligation and PCR. Following each step, with the exception of the adenosine base addition, the samples were cleaned using the TruSeq kit with Agencourt AMPure XP beads (Beckman-Coulter). 15 cycles of PCR were performed to amplify the selected fragments using PCR primers and a recipe supplied by Illumina. PCR products were quantitated with the Qubit fluorometer (Invitrogen). We loaded 3 ng PCR product per sample at a concentration of 5 pM onto the Illumina cBot cluster station for hybridization to the Illumina flowcell. The cBot performed bridge amplification to amplify single DNA molecules 28 times into clusters. Each cluster was then linearized, blocked, and the sequencing primer was hybridized. The flowcell was then ready to be loaded onto the HiSeq 2000. The flowcell was loaded and run with the Single Read 80 Base Pair Recipe on the HiSeq 2000, which allows sequencing of 73 single read bases plus 7 multiplexed bases. Illumina Real Time Analysis (RTA) within the instrument control software generated and analyzed images, produced base-call files, and ran quality scoring in real time. After sequencing of forward and reverse strands was complete, Illumina Casava software processed the data for quality analysis.

We performed downstream analysis using software developed internally. A correct sequence read should include 69 base-pairs (bp) of sequence, including the 5′ primer (20 bp), random region (29 bp) and 3′ primer (20 bp); any sequences not matching this pattern were filtered out. We allowed one mismatch in each primer during pattern matching. Primer sequences were trimmed out after filtering, leaving only the 29-bp aptamer sequences for downstream analysis. Reverse-complement tags were combined with the sequenced forward tags. The tag count was done at this stage, followed by round enrichment analysis.

We sequenced a total of four DNA aptamer pools from cell-SELEX R5 and R6, identifying 15 to 18 million sequence tags per pool (Table 1). Finally, we determined the ratios of aptamer tag numbers in the R6 c-kit pool versus parental cell pool (Table 2). Clustal Omega available on the EBI website (http://www.ebi.ac.uk/Tools/msa/clustalo/) was used to cluster the top 20 aptamer sequences listed in table 2 (Fig. S1) [29], [30].

### Flow Cytometry Analysis of Alexa-488-Labeled Aptamer Pools and Characterization of Individual Aptamers

We used an Accuri flow cytometer (BD Biosciences) for our aptamer binding studies because of its accuracy, large dynamic window and ease of use. We tested single-stranded DNA aptamer pools from cell-SELEX R5 and R6 for binding to BJAB c-kit cells and BJAB parental cells. In brief, the Alexa-488-labeled DNA aptamer pools were diluted to 200 nM in SELEX buffer, heated for 10 min at 95°C, snap-cooled on ice for 10 min and then mixed with 1.0×10^5^ cells of each cell line. After 60 min incubation on ice and two washes, mean fluorescence values of 5000 events per sample were read via flow cytometry and analyzed by CFLOW software (BD Biosciences).

We ordered individual selected c-kit aptamers as full-length 69-nt sequences from IDT. Each aptamer was synthesized with a 5′-biotin tag, which allowed detection with a streptavidin Alexa-647 conjugate. For the FACS assay, 2 µM aptamer stocks were prepared in SELEX buffer, heated for 10 min at 95°C, snap-cooled on ice for 10 min and kept at 4°C before testing. For initial binding studies, these aptamer stocks were further diluted to 200 nM in assay buffer (DPBS with Ca and Mg plus 0.1% BSA), mixed with equal volumes of BJAB-c-kit cells or BJAB parental cells to a final concentration of 100 nM and placed on ice. After one hour of incubation with occasional mixing, cells were washed by centrifugation and unbound aptamers were removed by aspiration. The washed cells were resuspended in a 1∶200 dilution of streptavidin-Alexa-647 conjugate (Life Technologies) and incubated for 30 min on ice. After two more washes in assay buffer, aptamer binding was measured in the Accuri. Individual aptamers that tested positive in this assay were further tested in serial dilutions from 0 to 100 nM. Aptamer candidate screening, at 100 nM, was performed in duplicate, and repeated (n = 3). Titration experiments were performed in singlicate and repeated. To determine statistical significance between group 1, BJAB-c-kit with Kit-129 at 100 nM and group 2, BJAB parental cells with Kit-129, mean fluorescence values were pooled and subject to students 2-tailed t-test analysis with a confidence interval of 95% (n = 3).

Using Graphpad Prism 6 and the equation: Y = B_max_X/(Kd+X), the apparent dissociation constant (K_d_) of the aptamer-cell interaction are obtained. For this calculation the mean-fluorescence background of controls is pre-subtracted [12].

### Aptamer Labeling of Mouse Bone Marrow

The femurs and tibias from two male C57BL/6J mice (Jackson Labs) were dissected and placed in ice-cold phosphate-buffered saline (PBS; Life Technologies). To isolate the marrow, we used a 23-gauge needle to bore a hole at both the proximal and distal ends of the hind limb bones. We then used a 25-gauge needle to pass ice cold FACS buffer (HBSS with 10 mM HEPES, 2% FBS and 0.1% sodium azide (Sigma) through the bone medulla, collecting the cellular flow-through in a 1.5 mL Eppendorf microcentrifuge tube. Alexa-488-labeled aptamers were diluted to 2 µM stock solutions, heated to 95°C for 10 minutes and snap-cooled on iced for 10 minutes. Whole mouse bone marrow was labeled in FACS buffer on ice for 30 minutes in the presence of 5 mM MgCl_2_ using combinations of 100 nM Alexa-488-labeled Kit-129 aptamer (IDT), fluorescein isothiocyanate (FITC)-labeled anti-mouse CD117 (c-kit clone 2B8, Biolegend) diluted 1∶100 and APC/Cy7-labeled anti-mouse CD45 (BD Biosciences) diluted 1∶100 in a v-bottom 96 well plate. Cells were analyzed by FACSCanto II using 488-nm and 635-nm lasers.

### Ethics Statement

All mice were maintained and handled in accordance with the recommendations in the Guide for the Care and Use of Laboratory Animals of the National Institute of Health. The protocol was also approved on 10/4/12 by the University of Wisconsin’s School of Medicine and Public Health institutional animal care and use committee (protocol code: M02059). All mice were euthanized according to protocol and all efforts were made to minimize suffering.

### Functional Studies of DNA Aptamer Kit-129

For the blocking experiment we used the Fluorokine Biotinylated Mouse SCF Kit (R&D Systems). We mixed BJAB c-kit cells with serial dilutions of Kit-129 in binding buffer, and then added biotinylated murine SCF-biotin to a final concentration of 100 ng/ml and incubated the mix on ice for 30 min. We added a 1∶200 dilution of streptavidin-Alexa-647 for the detection of bound biotinylated SCF, and incubated for 30 min on ice, washed and read mean fluorescence via flow cytometry. As a control, we used a Kit-129 aptamer with a scrambled core sequence (5′-ACG CGC CTA TGG TGC ATC GCA TGC CGA CC-3′).

### Binding of Kit-129 Aptamer to Mouse Mast Cell Line 11P0-1and BJAB-c-kit Cells

Cells were washed once in 500 µl of FACS buffer. 6-carboxyfluorescein (FAM)-labeled Kit-129 and scrambled control were heated to 95°C for 10 min and snap-cooled on ice for 10 min. We diluted 2 µM solutions of FAM-labeled Kit-129 and scrambled aptamer control (Kit-129SC) to generate a range of concentrations from 50 nM to 0 nM, and incubated these dilutions with BJAB, BJAB c-kit and 11P0-1 cells for 30 min on ice in FACS buffer plus 10 mM MgCl_2_. After 30 min, cells were washed once with FACS buffer and analyzed on the FACSCanto II using the 488-nm laser.

### Competition Assay with Anti-c-kit Antibody ACK2

We added Kit-129 aptamer to BJAB c-kit cells, then added ACK2–phycoerythrin (PE) (Novus Biologicals) to final concentrations of 100 nM and 100 ng/ml (∼1.8 nM) respectively in PBS 0.1% BSA, and incubated on ice for 1 hour. As a negative control, we used buffer alone, without Kit-129. We then analyzed ACK2-PE binding by flow cytometry.

### QPCR of Kit-129 Sorted Bone Marrow Populations

Whole mouse bone marrow was prepared and labeled as described above. Populations were sorted in FACS buffer using the BD FACS Aria III. The cells were spun down and resuspended into Trizol (invitrogen) for total RNA isolation. RNA was isolated from populations according to Trizol reagents manufactures instructions. Similar amounts of RNA from each population were used in cDNA synthesis using the cDNA synthesis VILO kit (Invitrogen). QPCR was performed using Taqman probes for mouse c-kit and Actin B on the Applied Biosytems’s ViiA7. Products were run on a 2% agarose gel.

## Supporting Information

Figure S1 Cluster analysis of the top 20 c-kit aptamers by Clustal Omega.
(TIF)

Figure S2 Dissociation curve of Kit-129 aptamer-cell interaction as analyzed by Graphpad Prism 6. BJAB c-kit cells were used as target expressing cells and parental BJAB cells were used as controls.
(TIF)
