# Supplementary figures and images for: Development of an Efficient Targeted Cell-SELEX Procedure for DNA Aptamer Reagents
Source: PLoS One. 2013 Aug 13;8(8):e71798. doi: 10.1371/journal.pone.0071798 (PMC3742456; doi:10.1371/journal.pone.0071798)

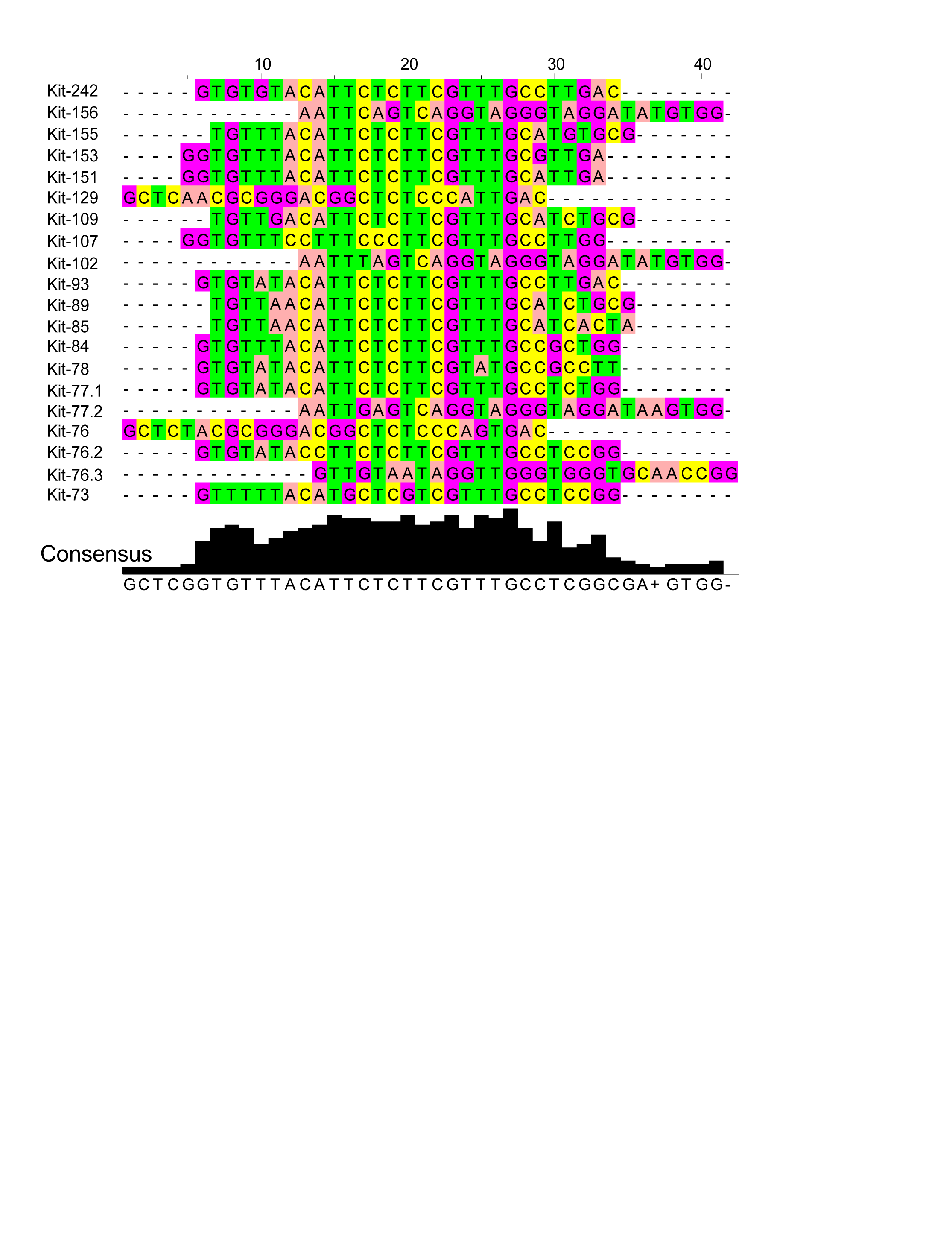

Supplement: Figure S1 — Cluster analysis of the top 20 c-kit aptamers by Clustal Omega. (TIF) [file pone.0071798.s001.tif]

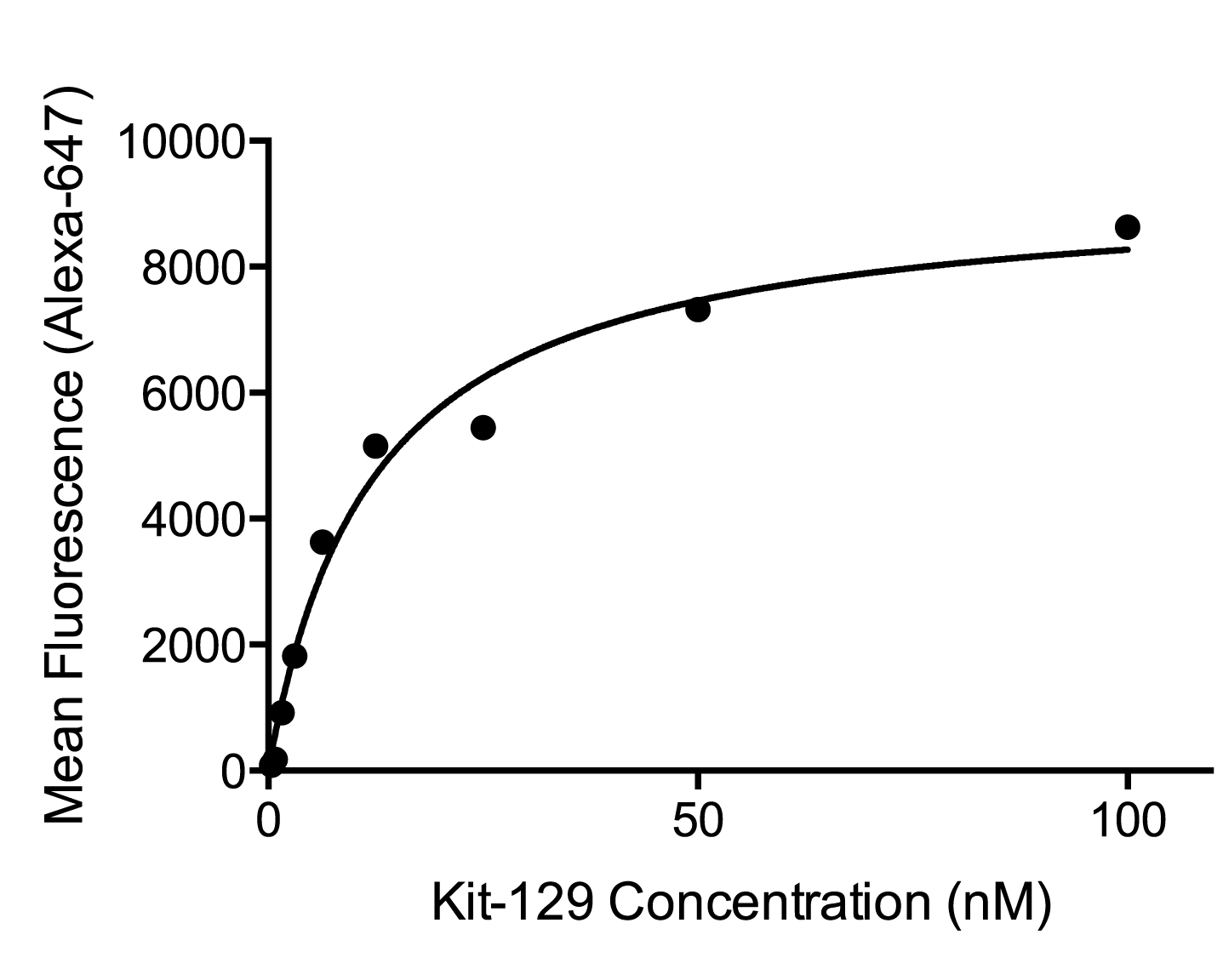

Supplement: Figure S2 — Dissociation curve of Kit-129 aptamer-cell interaction as analyzed by Graphpad Prism 6. BJAB c-kit cells were used as target expressing cells and parental BJAB cells were used as controls. (TIF) [file pone.0071798.s002.tif]
